# Preliminary Study of Australian Pinot Noir Wines by Colour and Volatile Analyses, and the Pivot© Profile Method Using Wine Professionals

**DOI:** 10.3390/foods9091142

**Published:** 2020-08-19

**Authors:** Rocco Longo, Wes Pearson, Angela Merry, Mark Solomon, Luca Nicolotti, Hanna Westmore, Robert Dambergs, Fiona Kerslake

**Affiliations:** 1 Horticulture Centre, Tasmanian Institute of Agriculture, University of Tasmania, Launceston-Prospect, Tasmania 7249, Australia; Angela.Merry@utas.edu.au (A.M.); Hanna.Wernstrom@utas.edu.au (H.W.); rodambergs@csu.edu.au (R.D.); Fiona.Kerslake@utas.edu.au (F.K.); 2 National Wine and Grape Industry Centre, Charles Sturt University, Wagga Wagga, New South Wales 2650, Australia; Wes.Pearson@awri.com.au; 3 The Australian Wine Research Institute, Adelaide, South Australia 5064, Australia; Mark.Solomon@awri.com.au (M.S.); Luca.Nicolotti@awri.com.au (L.N.); 4 Metabolomics South Australia, Adelaide, South Australia 5064, Australia; 5 WineTQ, Monash, South Australia 5342, Australia

**Keywords:** Australian Pinot noir, regionality, aroma compounds, Pivot© Profile, provenance

## Abstract

The aim of this preliminary study was to identify potential colour components, volatile and sensory attributes that could discriminate Pinot noir wines from five Australian winegrowing regions (Adelaide Hills, Yarra Valley, Mornington Peninsula, Northern and Southern Tasmania). The sensory analysis consisted of the Pivot© Profile method that was performed by wine professionals. A headspace solid-phase microextraction-gas chromatography-mass spectrometry method was used to quantify multiple volatile compounds, while the Modified Somers method was used for colour characterisation. Analysis of data suggested ethyl decanoate, ethyl 2-methylpropanoate, ethyl 2-methylbutanoate, in addition to decanoic acid as important contributors to the discrimination between regions. Similarly, wine hue, chemical age indices, total anthocyanin, and (%) non-bleachable pigment also discriminated wines between regions. The sensory analysis showed that wines from Mornington Peninsula were associated with the ‘red fruits’ aroma, ‘acidic’, and ‘astringency’ palate descriptors, while those from Adelaide Hills were associated with the ‘brown’ colour attribute. This study indicates regionality is a strong driver of aroma typicity of wine.

## 1. Introduction

Regionality (broadly referred to as terroir) is an important concept for winemakers and wine consumers, as it refers to how a wine is recognised on the basis of its geographical origin [1]. This is the result of the complex interplay between the grapevines and the surrounding environment (climate, soil, and site) in addition to viticultural and winemaking interventions that may affect or influence a genuine regional effect.

When dealing with the regional expression of Pinot noir wine, gas chromatography-mass spectrometry (GC-MS) and spectral analysis can be used in order to discriminate samples obtained from different geographical locations [2,3,4,5]. For example, a solid-phase extraction (SPE)-GC-MS method was used to discriminate Pinot noir wines from ‘high’- and ‘low’-typicity sites in New Zealand’s Central Otago [6], while ultra violet-visible (UV-Vis) spectrophotometry was used to discriminate Pinot noir wines from two sub-regions in the Chile’s Casablanca Valley [5].

As with aroma compounds and chromatic or colour structure components, relationships were hypothesised between the geographical origin of the grapes and the sensory features of the resulting wines. A descriptive analysis (DA) of 28 Pinot noir wines from California showed that samples from the cool Carneros appellation were more intense in the ‘fresh berry’, ‘berry jam’, ‘cherry’, and ‘spicy’ descriptors than those from the warmer Napa and Sonoma Valleys [7]. Descriptive analysis was also used to differentiate 32 Pinot noir wines from New Zealand, with samples from Marlborough receiving higher scores than those from Waipara and Martinborough for the ‘red cherry’ and ‘raspberry’ aromas, for example [8].

Descriptive analysis is a very robust and reliable sensory approach. However, it can be time consuming and costly, as panellists require extensive screening and training for up to several months [9]. A more recent and rapid sensory discriminant technique is the Pivot© profile, where panellists are presented with a known sample called ‘pivot’ as a reference for evaluating unknown samples [10]. The panellists are then required to generate an unlimited number of sensory descriptors based on how the unknown sample differs from the pivot. The format of this approach is to include any descriptor the panellists generate in conjunction with ‘less than’ or ‘more than’ the pivot. Product category experts, such as winemakers or wine judges, are recommended when using Pivot© profile, as they already have a pre-existing lexicon available [11].

Pinot noir (*Vitis vinifera* L.) is a very popular cultivar, particularly in cool-climate wine growing regions, where it produces fine, elegant, and expressive wines [12]. This red grape variety accounts for over 112,000 ha of vines planted around the world [13] and the wines are generally associated with a range of aromas, from simple attributes, such as ‘floral’ and ‘red fruits’, to more complex, such as ‘earthy’, ‘humus’, ‘mushroom’, and ‘liquorice’ [14].

Pinot noir accounts for over 5000 ha of vineyards from across Australia. Australia has a very old tradition with Pinot noir, since it was first planted in the 1830s by James Busby. Today, it is the second most popular red cultivar amongst consumers with a 23% share of the ‘red wine consumed in domestic on-trade’ category behind Shiraz with a share of 31% [15].

The aim of this preliminary study was to identify colour, volatile, and sensory attributes that could help to discriminate Pinot noir wines from different Australian geographical origins. The wines were sourced from five regions where this cultivar is locally relevant, namely Adelaide Hills in South Australia, Yarra Valley, and Mornington Peninsula in Victoria, Southern, and Northern Tasmania. The findings of this preliminary study may help the Australian wine industry understand how Pinot noir performs in their region and then use this to promote their wines and educate their consumers.

## 2. Materials and Methods

### 2.1. Selected Wines

The set of 15 wines examined in this study included three representative Pinot noir wines from five Australian regions (Adelaide Hills, Yarra Valley, Mornington Peninsula, Northern and Southern Tasmania). The wines were selected after consultation with noted Australian winemakers and wine judges (Table 1). The primary selection criterion was that the wine was from a single vineyard to minimise any confounding effect that originates from blending components from other regions. The pivot wine was agreed upon by a group of wine experts highly experienced in Pinot noir wine sensory properties. It was an unoaked, multi-regional Tasmanian Pinot noir wine without any strong, individualistic sensory characters. The wines selected were examined by experienced sensory scientists prior to the sensory analysis and none of them presented organoleptic faults. All of the wines were sealed with screwcaps and stored at room temperature (20 °C).

#### 2.1.1. Basic Oenological Attributes

The alcohol levels were commercially available and, thus, are expected to be ±0.5%, as per regulations. Titratable acidity (TA) and pH were determined by sodium hydroxide titration to an end point of pH 8.20 with a Fully Automated 59 Place Titrando System (Metrohm, Herisau, Switzerland).

#### 2.1.2. Vineyard Sites

The wines were sourced from 15 different vineyard sites, in general, on own-roots, drip irrigated, and Vertical Shoot Positioned (i.e., vine shoots were trained upward in a vertical curtain with the fruiting zone below).

Australian geography is very heterogenous. This results in a range of climatic zones with many cool climate regions that are influenced by the Pacific Ocean and others influenced by the Tasman Sea. The main climate parameters for the growing season October 2017–April 2018, together with the altitude, for each of the five Australian regions examined in this study are summarised in Table 2. They include: (a) the cumulative growing degree-days (GDDs) (base 10 °C) from 1 October 2017 to 30 April 2018; (b) the average growing season temperature (GST) from 1 October 2017 to 30 April 2018; (c) the mean monthly temperature for January and February 2018; and, (d) the total rainfall from 1 October 2017 to 30 April 2018. The data were retrieved from proximal Bureau of Meteorology sites that were available from the Queensland Government’s online database SILO (https://www.longpaddock.qld.gov.au/silo/).

### 2.2. Pivot© Profile

A panel of 11 wine professionals (eight males and three females aged 35–55 years) who regularly participate in wine shows or tasting events were convened (in January 2020) to evaluate the samples while using the Pivot© profile sensory method [10]. The panellists were recruited from Tasmania (with the exception of two panellists from South Australia) via email and selected on the basis of their availability and willingness to participate to the project. None of them had previous experience with this approach. Each panellist was presented with 50 mL of each wine and 100 mL of pivot wine, with more available if requested. The wines were served in Riedel Ouverture Red wine glasses, marked with three-digit codes. The wines were presented in a randomised order at ambient temperature (20 ± 2 °C). The Pivot© Profile analysis was performed in an open plan room on tables with all samples presented at once, plus the pivot wine (*n* = 16 in total). Data were collected on personal digital tablets using the Compusense sensory software (Compusense Inc., Guelph, Canada). Panellists generated appearance, aroma, and palate attributes to be compared against the pivot, as described in Thuillier et al. [10]. Social science ethics approval for the collection of tasting data was obtained from the University of Tasmania’s Research Integrity and Ethics Unit (Ref No: H0015927).

### 2.3. Headspace Solid-Phase Microextraction-Gas Chromatography-Mass Spectrometry

#### 2.3.1. Esters, Alcohols and Fatty Acids

A total of 28 fermentative compounds were analysed (in January 2020) by solid phase micro extraction (SPME)-GC-MS according to previously published method using Agilent Technologies Ltd. equipment (Melbourne, Australia) [16]. One mL of each sample was pipetted into 20 mL SPME vials with 9 mL of saturated potassium hydrogen tartrate buffer (pH 3.7) and 2 g of NaCl. An Agilent 7890A GC that was equipped with a Gerstel MPS2 multi-purpose sampler and coupled to an Agilent 5975C VL mass selective detector was used to perform the analysis. The GC was fitted with an Agilent DB-624UI column (30 m × 0.25 mm, 1.4 μm film thickness) with He as the carrier gas. The oven was started at 40 °C, increased to 60 °C at 20 °C min.^−1^ (held for 14 min.) and then followed by series of temperature ramps. First ramp to 80 °C at 10 °C min.^−1^, second ramp to 160 °C at 20 °C min.^−1^, and third ramp to 260 °C at 10 °C min.^−1^ and held for 2 min. for a total run time of 45.5 min. The SPME vials and its contents were heated to 40 °C for 5 min. with agitation. The SPME fibre was exposed to the headspace in the sample for 15 min. and then desorbed in the injector (splitless mode) for 15 min. The injector was set at 260 °C. The MS quadrupole was set at 150 °C, the source was set at 230 °C, and the transfer line was held at 260 °C. Positive ion electron impact spectra at 70 eV were recorded in SIM and SCAN mode with a solvent delay of 4 min.

The raw data from Agilent’s ChemStation software (ver. E.02.02.1431) were processed by the MassHunter Workstation Software for Quantitative Analysis for GC-MS (ver. B.09.00). A stable isotope dilution analysis (SIDA) was used to determine the concentration of analytes in the samples. All of the target and qualifier ions of internal standards and analytes, in addition to their expected retention time, are reported in Supplementary Table S1.

#### 2.3.2. C_13_-Norisoprenoids

The C_13_-norisoprenods α-ionone, β-ionone, and β-damascenone were analysed (in January 2020), as follows. Ten mL wine sample was transferred into 20 mL SPME vials with 2 g of NaCl and 50 µL of a combined d_4_-β-damascenone, d_3_-α-ionone, and d_3_-β-ionone internal standard solution. The GC-MS analysis was performed on an Agilent 7890 GC that was equipped with a Gerstel MPS2 autosampler and coupled to an Agilent 5977B N mass selective detector. The GC was fitted with an Agilent DB-5 MS (30 m × 0.25 mm, 0.25 µm film thickness) and ultra-high purity He was the carrier gas. The oven was started at 40 °C, held at this temperature for 1 min. then increased to 190 °C at 8 °C min.^−1^ and held at this temperature for 5.25 min. The vials and its contents were heated to 60 °C for 10 min. in the heater/agitator. The SPME fibre was exposed to the sample during this heating time and injected into a split/splitless inlet in splitless mode. The analytes were desorbed into a Supelco 0.75 mm ID sleeveless SPME liner (Supelco, Bellefonte, PA, USA) for 10 min., which was held at 200 °C. The purge flow to the split vent was 50 mL min^−1^ at 2.1 min. with the septum purge flow turned off. The mass spectrometer quadrupole temperature was set at 150 °C, the source was set at 230 °C, and the transfer line was held at 250 °C. EMV Mode was set to Gain Factor = 1.00 and th spectra were recorded in SIM mode. The ions monitored in SIM mode were: m/z 73, 179 and 194 for d_4_-β-damascenone and m/z 69, 175, and 190 for β-damascenone; m/z 112, 139, and 195 for d_3_-α-ionone and m/z 109, 136, and 192 for α-ionone; m/z 180, 181, and 195 for d_3_-β-ionone, and m/z 177, 178, and 192 for β-ionone.

### 2.4. Chromatic Components and Colour Structure Analysis

The Pinot noir wine samples were analysed (in January 2020) according to the Modified Somers Assay, as previously described in Mercurio et al. [17]. The wine samples were degassed and centrifuged at 3500 rpm for 15 min. using a 5804 Eppendorf (Hamburg, Germany). They were then added to four different buffers as follows: (1) 1:10 dilution of wine in buffer 1 (model wine, 0.5% *w*/*v* tartaric acid in 12% *v*/*v* ethanol adjusted to pH 3.4 with 5 M NaOH); (2) 1:10 dilution of wine in buffer 1 plus 0.375% *w*/*v* sodium metabisulphite; (3) 1:10 dilution of wine in buffer 1 plus 0.1% *v*/*v* acetaldehyde; (4) 1:50 dilution of wine in 1 M HCl. The samples were mixed and incubated at room temperature (20 °C) in the dark for at least 1 h and then read with a GENESYS 10S UV-Vis single cell Spectrophotometer (Thermo Fisher Scientific, Waltham, MA, USA) using the following wavelengths: 250, 270, 280, 290, 315, 420 and 520 nm. All of the parameters were determined from spectral data according to the calculations that were outlined in Mercurio et al. [17].

### 2.5. Data Analysis

All of the data sets were analysed by one-way Analysis of Variance (ANOVA) using R (R Core Team, Vienna, Austria). Equal variances of regional groups were verified by using Levene’s Test. Duncan’s multiple range-test at *p* = 0.05 was used to test the differences between regional means. Principal component analysis (PCA) was performed with JMP (ver. 14, SAS Institute, Cary, NC, USA). Separate PCA was performed for volatile and colour data sets. PCAs were developed using only the significant parameters (*p* < 0.05) to avoid fitting noise to the models.

Raw Pivot© Profile results were downloaded into a spreadsheet and organized by ‘more than’ and ‘less than’ terms. Some lemmatisation was required during this analysis in order to reconcile terms that have similar meanings, for example ‘tannin’, ‘tannic’, ‘blocky tannin’, ‘tannins’, ‘soft tannin’, ‘hard tannin’ were all grouped under the one term: ‘tannin’. A data matrix was then created, with frequencies of the ‘less than’ terms being subtracted from the ‘more than’ terms. As this leaves some values negative (some attributes would be used as ‘less than’ more than ‘more than’), the results were adjusted to contain only positive values, with the most negative value added to complete set, leaving the most negative attribute zero and the remaining attribute’s positive. The results were then analysed by correspondence analysis (CA) using XLStat (Addinsoft, Paris, France) to give a biplot of the samples and sensory attributes. Analysis was performed on appearance, aroma, and palate attributes individually, and then another with terms deemed to be relevant in separating the wines. This was determined by standard deviation of the attributes in the frequency table. Attributes with a standard deviation > 1.5 were included in the final CA biplot. In this instance, setting the minimum standard deviation at 1.5 excluded many of the fringe attributes and minimises noise that would have otherwise cluttered the analysis. For this analysis, the original appearance, aroma, and palate data were normalised to have the most negative score from all three modalities equal zero.

## 3. Results and Discussion

### 3.1. Volatile Analysis

A total of 31 analytes were identified and quantified in the headspace of 15 commercial, single-vineyard Australian Pinot noir wines. Table 3 reports the mean concentration of each compound by region, together with their odour thresholds, and aroma descriptors. The odour activity value (OAV) of each compound, calculated as the ratio of the concentration to the odour detection threshold, is also presented in Table 3. Hexanoic acid, hexyl acetate, propanoic acid, and acid 2-phenyl acetate were excluded from Table 3, as they were found to be below their limit of quantification.

Apart from acetic acid, which was found in the range of 600 to 700 mg L^−1^, the most abundant volatile compounds were the higher alcohols, such as 3-methylbutanol, 2-methylbutanol, and 2-methylpropanol. Higher alcohols are important contributors to the complexity of wine aroma. However, they give wines ‘solvent’ and ‘fusel’ characters at concentrations greater than 400 mg L^−1^, which may mask more elegant aromas that are associated with ethyl esters [18]. On the basis of the ANOVA results, four volatile compounds (i.e., ethyl decanoate, ethyl 2-methylpropanoate, ethyl 2-methylbutanoate, and decanoic acid) appeared to significantly contribute to the discrimination between regions (Table 3).

Ethyl esters of fatty acids such as ethyl decanoate are yeast’ metabolism by-products, and their concentrations in wines largely depend on winemaking conditions (e.g., selected yeast, fermentative temperature) [19]. By contrast, ethyl esters of branched acids, such as ethyl 2-methylpropanoate and ethyl 2-methylbutanoate, are primarily formed during wine aging by esterification between branched acids and ethanol [19]. However, they can also originate from yeast during alcoholic fermentation through branched amino acid metabolism [20].

There is little information in the literature with regards to the discrimination of Pinot noir wine on the basis of grape geographical origin and volatile compounds [21]. Where the effect of vineyard site was evaluated on the volatile profile of Pinot noir wine, this effect was mostly vintage dependent, and only β-citronellol, homovanillyl alcohol, *N*-(3-methylbutyl)acetamide, and *N*-(2-phenylethyl)acetamide discriminated the vineyard sites independent of vintage [6]. These authors also showed that Central Otago’s Pinot noir wines could be discriminated on the basis of the geographical origin, thanks to the different ratio in higher alcohols, ethyl esters, and acetate esters [4].

As reported in Table 3, the samples from Yarra Valley had a higher concentration in ethyl decanoate then those from the Mornington Peninsula and Northern Tasmania. Ethyl decanoate is an important compound for Pinot noir wine [8]. High concentrations of ethyl decanoate in combination with ethyl octanoate enhanced a ‘black cherry’ aroma of reconstituted Pinot noir wine, while, in combination with 2-phenyl ethanol, influenced the ‘jam’ and ‘smoky’ aromas [22]. Ethyl 2-methylbutanoate and ethyl 2-methylpropanoate, which significantly changed in this study, were previously suggested as contributors to the aroma of Pinot noir wine by means of aroma extraction dilution analysis (AEDA) [2,23]. While ethyl 2-methylbutanoate was associated by these authors to ‘fruity’, ‘resin’, ‘honey’, and ‘sweet’ notes, ethyl 2-methylpropanoate was described as having ‘sweet’, ‘fruity’, and ‘apple’ aromas. The samples from Northern Tasmania had higher concentrations in ethyl 2-methylbutanoate than the wines from Adelaide Hills. Likewise, the samples from Northern Tasmania had a higher concentration in ethyl 2-methylpropanoate than those from Adelaide Hills, Yarra Valley, and Southern Tasmania. Noteworthy, ethyl 2-methylpropanoate was found in the range of 121–255 µg L^−1^ and OAVs of 8–17. OAVs > 1 should theoretically have an impact on the aroma, although compounds with 1 > OAV > 0.5 have been shown to add wines complexity [24]. It could be possible that ethyl 2-methylpropanoate plays an important role to the aroma of Australian Pinot noir wines.

Decanoic acid was the only fatty acid to significantly change between regions (Table 3). Specifically, its concentration was higher in wines from Yarra Valley as compared to those from Adelaide Hills, Mornington Peninsula, and Northern Tasmania (Table 3). At levels near odour thresholds, fatty acids add ‘complexity’ to wine; but, at higher concentrations, they can impart off-odours reminiscent of ‘sweat’ [18]. In this study, decanoic acid was found in the range of 336–661 µg L^−1^, which is well below its odour threshold of 1000 µg L^−1^ (determined in 11% v/v aqueous ethanol with 7 g L^−1^ glycerol, at pH 3.2) [25]. Despite decanoic acid having an OAV < 1, its contribution to the overall aroma of these wines cannot be excluded [26].

### 3.2. Chromatic and Colour Structure Analysis

We quantified a number of chromatic and colour structure components in order to discriminate Australian Pinot noir wines on the basis of their geographical origin. The results for the 15 wine samples are summarised in Table 4. Of all parameters, hue, ‘chemical age’ 1 and 2, total anthocyanin, and (%) non-bleachable pigment were significantly different (*p* < 0.05) between regions.

The concentration of total anthocyanins (expressed as mg L^−1^ malvidin 3-O-glucoside) was significantly different between regions, with the wines from Southern Tasmania and Yarra Valley having higher levels than those from Mornington Peninsula (Table 4). Anthocyanins in Pinot noir grapes only exist in their less stable, non-acylated, glycosidically-bound forms that are very reactive [30]. They can either be lost through oxidation or converted into more stable colour forms by reacting with tannin [31]. The reaction of anthocyanins with tannin is influenced by several factors, such as sulfur dioxide levels, pH, oxygen uptake, and even the strain of yeast winemakers use or whether they do wild ferments [32]. A combination of these factors may explain the different wine anthocyanin concentrations between regions.

Hue values and (%) non-bleachable pigment (i.e., SO_2_-resistant) were higher in the wines from Adelaide Hills and Mornington Peninsula when compared to those from Yarra Valley, Southern and Northern Tasmania (with the exception of % non-bleachable pigment, which was not significantly different between Adelaide Hills and Mornington Peninsula with Northern Tasmania) (Table 4). Hue (or tonality) describes the progressive change in a wine from deep purple red towards red-brick, and then brown colour [33]. Hue was calculated as the ratio of 420 nm (yellow) to 520 nm (red) absorbance [17]. While the absorbance at 420 nm relates to tannin and anthocyanin reaction products, which, at 520 nm, is associated with free anthocyanins in the flavilium cation form and anthocyanins-tannins combinations. Hue values increase with ageing, as shown for other Pinot noir wines a 24-month in-bottle storage in the dark at 18 °C [34]. Similarly, ‘chemical age’ 1 and 2 were higher in the wines from Adelaide Hills than those from Yarra Valley, Northern and Southern Tasmania (with the exception of ‘chemical age’ 2, which was not significantly different between Adelaide Hills and Northern Tasmania wines). ‘Chemical age’ 1 was calculated as the ratio of 520 nm-sulfite absorbance to 520 nm-acetal absorbance, while ‘chemical age’ 2 is equivalent to the ratio of 520 nm-sulfite absorbance to 520 nm-clorhidric absorbance multiplied by 5. Wine ‘chemical age’ indexes describe the increase in the wine colour of oligomeric and polymeric pigments that progressively become less susceptible to pH changes and to bleaching by dioxide sulfide [35]. These findings suggest that the Pinot noir wines from Adelaide Hills develop a brick-red/brown colour quicker than those from the other regions. Because hue is a very sensitive measure of the reaction of anthocyanins (they are more purple and have low hue values) and tannins (they are more brick red/brown so have a high hue value), winemaking effects, such as oxygen uptake during the first stages of the fermentation [36], or use of different yeast [32], may explain these results. Additionally, the Adelaide Hills wines had the highest pH (Table 1) and there may be some regional differences in tannin extractability related to climate that may drive accelerated formation of pigmented tannins [37].

### 3.3. Principal Component Analysis

Non-supervised pattern recognition statistical analysis was employed through principal component analysis (PCA). The approach taken was not to generate a predictive model that could be applied to unknown samples, due to the experimental design being limited to three wines per five regions only. Rather, the aim was to identify possible patterns that were related to the classification factors.

**Figure 1:**
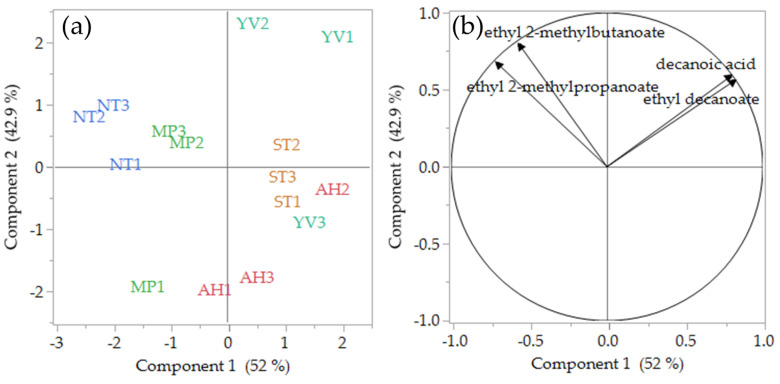
Principal component analysis (PCA) scores (**a**) and loadings (**b**) plots of 2018 Pinot noir wines (*n* = 3 per region) and significant (*p* < 0.05) volatile compounds. AH, Adelaide Hills; MP, Mornington Peninsula; YV, Yarra Valley; ST, Southern Tasmania; NT, Northern Tasmania.

Figure 1a,b illustrate the PCA scores (a) and loadings (b) plots of 2018 Pinot noir wines built on the volatile analysis results. Figure 1a shows a plot of the first two components, which explained over 95% of the total variability. Notably, all of the volatile compounds are loaded into the upper quadrants (Figure 1b). As illustrated by Figure 1a, samples from Northern Tasmania (NT) and Mornington Peninsula (MP) had negative values for Component 1 (PC1), while those from Southern Tasmania (ST), Yarra Valley (YV), and Adelaide Hills (particularly AH3 and AH2) samples had positive values. These preliminary results, in agreement with the ANOVA results reported in Table 3, suggest some common patterns in terms of volatile composition between wines of the same region, at least for AH1 and AH3, YV1 and YV2, MP2 and MP3, and NTs and STs. These findings are even more relevant if we consider the wines used in this study were all commercial wines, and only AH3 and ST1 were produced from the same winemaker.

**Figure 2:**
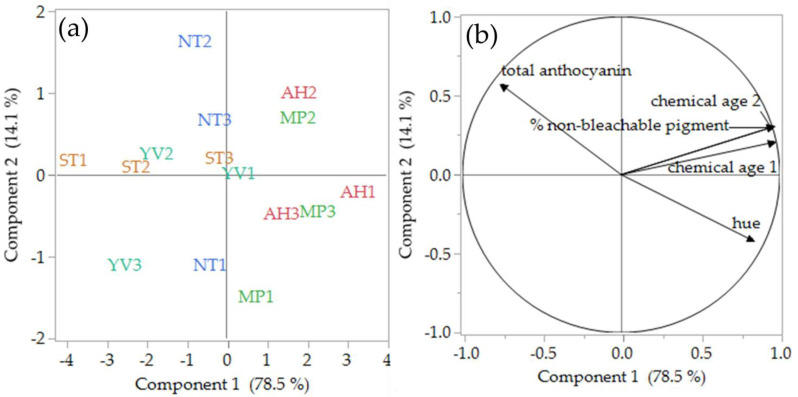
PCA scores (**a**) and loadings (**b**) plots of 2018 Pinot noir wines (*n* = 3 per region) and significant (*p* < 0.05) chromatic and colour structure attributes. Adelaide Hills; MP, Mornington Peninsula; YV, Yarra Valley; ST, Southern Tasmania; NT, Northern Tasmania.

Figure 2a,b illustrate PCA scores and loadings plots of 2018 Pinot noir wines based on the colour measurement results. The first two components explain over 93% of the total variability, with the separation of the regions driven predominantly by PC1. Apart from the Northern Tasmania (NT), ST3 and YV1 samples, which were neutral on PC1 (Figure 2a), the rest of the samples had positive or negative scores. Samples from Southern Tasmania (ST) and Yarra Valley (particularly YV2 and YV3) had negative scores for PC1, representing the higher total anthocyanin content. On the other hand, the samples from Adelaide Hills and Mornington Peninsula had positive scores for PC1 (Figure 2a), which suggests they had more developed colour attributes, such as high hue and ‘chemical age’ 1, in agreement with the ANOVA results that are reported in Table 4.

### 3.4. Sensory Analysis

**Figure 3:**
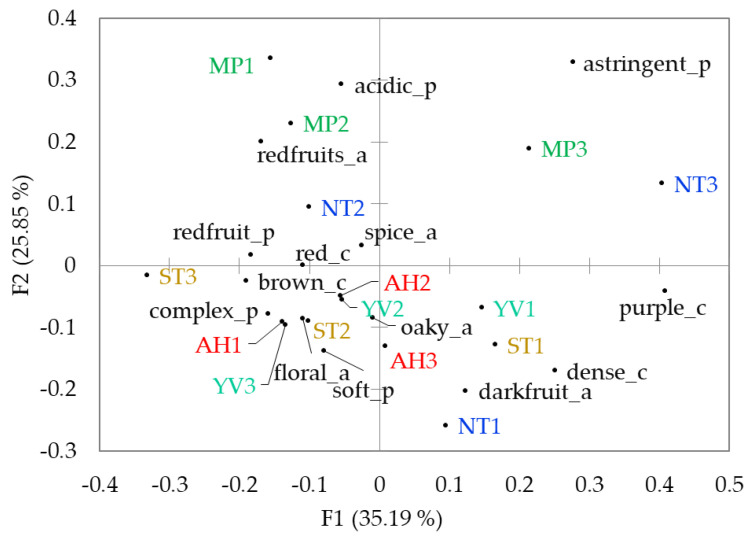
Correspondence analysis biplot of the 15 Pinot Noir wines using Pivot© Profile. AH, Adelaide Hills; MP, Mornington Peninsula; YV, Yarra Valley; ST, Southern Tasmania; NT, Northern Tasmania; c: colour attributes, a: aroma attributes, p: palate attributes.

A total of 53 descriptors were generated by a panel of wine professionals (e.g., winemakers, wine judges): 10 appearance, 22 aroma, and 21 palate. The generated descriptors were analysed individually. Subsequently, the attributes that had a standard deviation < 1.5 in the frequency matrix (Supplementary Table S2) were included in the final correspondence analysis (CA), which left 14 attributes (four appearance, and five for both aroma and palate) (Figure 3). CA is a variant of PCA that applies to categorical rather than continuous data and is generally used to analyse Pivot© profile data [10].

Combining all of the most important attributes from the appearance, aroma and palate biplot provides an overall characterisation of the Pinot noir samples (Figure 3). F1 and F2 combine to explain approximately 61% of the variance, with F3 explaining 11%, and F4 explaining another 9% (data not shown). Trends in the sensory profile by region are apparent with the biplot that is illustrated in Figure 3. Two samples from Southern Tasmania (ST2, ST3), two from the Adelaide Hills (AH1, AH2) and two from the Yarra Valley (YV2, YV3) are reasonably tightly grouped together with the ‘lower-left’ quadrant variables. These groups appear to be based on the ‘brown’ colour, ‘complex’ and ‘soft’ palate, ‘floral’ and ‘oaky’ aromas. The association between Adelaide Hills samples with the ‘brown’ colour attribute is consistent with the results that were obtained from ANOVA (Table 4) and PCA (Figure 2), confirming that wines from this region age quicker than the others. By contrast, the Mornington Peninsula samples grouped together into the upper quadrants, based on ‘red fruits’ (as aroma descriptor) and ‘acidic’. The ‘astringent’ descriptor was also associated with the wines from Mornington Peninsula, particularly with MP3. Astringency is a tactile sensation and it originates from binding and subsequent precipitation of tannins with salivary proteins and glycoproteins [38]. Although the concentration of tannin was not significantly different between regions, its value was much higher for the Mornington Peninsula samples in comparison to the rest of the regions (Table 4), perhaps at a level higher enough to be perceived by the panel.

The wines from Northern and Southern Tasmania are spread out across the biplot exhibiting sensory characters that cover the broad spectrum of sensory attributes used to describe these Pinot noirs. For example, ST1, NT1, and YV1 seem, to some extent, to agree well with the ‘lower-right’ variables (i.e., ‘dark fruit’ aroma, ‘dense’, and ‘purple’ colour). Likewise, NT3 and MP3 agree well with the ‘astringent’ descriptor in the ‘upper-right’ quadrant. This is possibly due to the bigger geographical area of the Northern and Southern Tasmania regions as compared to the other regions, responsible for diluting a true regional effect.

## 4. Conclusions

This preliminary study shows there are some regional chemical and sensory distinctiveness when it comes to Australia’s Pinot noir producing regions. From the volatile analysis, ethyl decanoate, ethyl 2-methylpropanoate, ethyl 2-methylbutanoate, and decanoic acid can be proposed as potential markers, suggesting that fermentation plays a significant role in the regional typicity of Australian Pinot noir wine. Some similarities were also apparent in terms of chromatic components and colour structure, in particular for the Adelaide Hills samples, which appeared to age more quickly than other regions.

Some sensory similarities among wines from the same region were observed despite wines likely being produced using different winemaking techniques. In particular, wines from the Mornington Peninsula (and to a lesser extent those from Northern Tasmania) were associated with the ‘red fruits’ aroma, ‘acidic’, and ‘astringent’ palate descriptors, while the majority of wines from Adelaide Hills, Southern Tasmania, and Yarra Valley was associated with the ‘brown’ colour, ‘complex’ and ‘soft’ palate, ‘floral’ and ‘oaky’ aromas. This information, although preliminary, is important for those wine characters promoting regional typicity. Further analysis of a larger number of commercial samples and, more importantly, wines produced under standardised winemaking protocols is warranted to better understand this relationship.

## Figures and Tables

**Table 1:** Regional means (± standard deviation, SD) of basic oenological attributes of the sample set of Pinot noir wines used (*n* = 3 per region).

Region	State	Vintage	Alc. % (*v*/*v*)	pH	Titratable Acidity (g L^−1^)
Adelaide Hills	SA	2018	13.8 ± 0.6	3.9 ± 0.2	5.14 ± 0.35
Yarra Valley	VIC	2018	13.4 ± 0.3	3.7 ± 0.1	5.43 ± 0.36
Mornington Peninsula	VIC	2018	13.8 ± 0.3	3.7 ± 0.1	5.88 ± 0.18
Northern Tasmania	TAS	2018	13.3 ± 0.3	3.6 ± 0.2	5.83 ± 0.51
Southern Tasmania	TAS	2018	13.6 ± 0.2	3.8 ± 0.1	5.56 ± 0.28

SA, South Australia; VIC, Victoria; TAS, Tasmania.

**Table 2:** Altitude, and means (±SD) of climatic data for the viticultural sites (*n* = 3 per region) from 1 October 2017 to 30 April 2018 (i.e., growing season in the Southern Hemisphere).

Region	State	Altitude (m a.s.l.)	GDD (days) ^1^	GST (°C) ^2^	January (°C) ^3^	February (°C) ^4^	Rainfall (mm) ^5^
Adelaide Hills	SA	340–540	1923 ± 117	19.1 ± 0.6	22.3 ± 0.6	21.5 ± 0.6	236 ± 33
Yarra Valley	VIC	150–210	1766 ± 27	18.3 ± 0.2	21.4 ± 0.2	20.8 ± 0.2	453 ± 17
Mornington Peninsula	VIC	90–240	1697 ± 70	18.0 ± 0.3	20.7 ± 0.4	20.2 ± 0.4	348 ± 11
Northern Tasmania	TAS	25–150	1303 ± 29	16.1 ± 0.1	19.0 ± 0.2	17.9 ± 0.2	318 ± 24
Southern Tasmania	TAS	<50	1210 ± 133	15.7 ± 0.6	18.4 ± 0.6	16.4 ± 0.5	317 ± 56

SA, South Australia; VIC, Victoria; TAS, Tasmania; ^1^ The total mean monthly growing degree-days (GDDs) (base 10 °C) from 1 October 2017 to 30 April 2018 (mean of *n* = 3 vineyard sites); ^2^ the growing season average temperature (GST) from 1 October 2017 to 30 April 2018 (mean of *n* = 3 vineyard sites); ^3^ the average monthly temperature for January 2018 (mean of *n* = 3 vineyard sites); ^4^ the mean monthly temperature for February 2018 (mean of *n* = 3 vineyard sites); ^5^ the total rainfall from 1 October 2017 to 30 April 2018 (mean of *n* = 3 vineyard sites).

**Table 3:** Means (µg L^−1^) (±SD) of compound concentrations in 2018 Pinot noir wines (*n* = 3 per region) with associated odour thresholds and aroma descriptors.

Analyte	Odour Threshold (µg L^−1^) and Descriptor/s	Adelaide Hills	Yarra Valley	Mornington Peninsula	Northern Tasmania	Southern Tasmania	Odour Activity Value (min–max)	*p*
**Higher Alcohols**								
2-Methylpropanol (mg L^−1^)	40 ^1^ (mg L^−1^) (solvent)	69 ± 20	66 ± 3	75 ± 8	100 ± 38	78 ± 15	1.6–2.5	ns
3-Methylbutanol (mg L^−1^)	30 ^1^ (mg L^−1^) (solvent)	168 ± 20	183 ± 34	176 ± 33	254 ± 68	197 ± 29	5.6–8.5	ns
2-Methylbutanol (mg L^−1^)	1.2 ^2^ (mg L^−1^) (solvent)	80 ± 7	94 ± 17	97 ± 12	130 ± 40	105 ± 16	67–108	ns
2-Phenylethanol (mg L^−1^)	10 ^1^ (mg L^−1^); 14 ^3^ (mg L^−1^) (floral)	21 ± 8	22 ± 7	24 ± 7	29 ± 4	19 ± 5	1.9–2.9; 1.3–2.1	ns
1-Hexanol	8000 ^1^ (cut grass)	1829 ± 595	1629 ± 297	1622 ± 770	1968 ± 779	2350 ± 924	0.20–0.29	ns
Butanol	150,000 ^2^ (fusel)	1789 ± 894	1648 ± 259	1282 ± 123	1302 ± 127	2122 ± 1016	0.01	ns
Total (mg L^−1^)	-	342 ± 56	368 ± 61	375 ± 61	516 ± 151	403 ± 67	-	ns
**Ethyl Esters**								
Ethyl propanoate	9000 ^2^ (fruity)	205 ± 40	189 ± 56	170 ± 18	183 ± 18	177 ± 35	0.02	ns
Ethyl butanoate	20 ^1^ (acid fruit, apple)	238 ± 90	272 ± 4	166 ± 43	196 ± 41	258 ± 88	8.3–12.9	ns
Ethyl hexanoate	5 ^1^; 14 ^3^ (green apple)	346 ± 121	415 ± 46	257 ± 75	326 ± 26	431 ± 74	51–86; 18.3–30.8	ns
Ethyl octanoate	2 ^1^, 5 ^3^ (sweet, fruity)	384 ± 114	495 ± 27	300 ± 117	350 ± 16	453 ± 83	150–247; 60–99	ns
Ethyl decanoate	200 ^3^ (grape)	140 ± 61 abc	218 ± 52 a	100 ± 38 bc	89 ± 12 c	182 ± 35 ab	0.44–1.09	<0.05
Ethyl 2-methylpropanoate	15 ^1^ (sweet, fruity)	121 ± 18 c	178 ± 54 bc	198 ± 28 ab	255 ± 40 a	149 ± 30 bc	8.1–17.0	<0.01
Ethyl 2-methylbutanoate	1 ^1^; 18 ^3^ (apple)	8.4 ± 1.1 b	14.1 ± 6.3 ab	14.1 ± 4.4 ab	18.6 ± 1.0 a	11.2 ± 0.1 b	8.4–18.6; 0.47–1.03	<0.05
Ethyl 3-methylbutanoate	3 ^3^ (fruity)	11.8 ± 1.9	21.2 ± 9.3	20.6 ± 8.3	27.7 ± 4.0	15.0 ± 0.8	3.9–9.2	ns
Total (µg L^−1^)	-	1454 ± 447	1802 ± 255	1226 ± 332	1445 ± 158	1676 ± 346	-	ns
**Acetate Esters**								
Ethyl acetate (mg L^−1^)	12.2 ^4^ (mg L^−1^) (fruity)	98 ± 13	102 ± 27	102 ± 14	88 ± 6	91 ± 18	7.2–8.3	ns
2-Methylpropyl acetate	1600 ^2^ (fruity)	61 ± 6	62 ± 19	68 ± 12	79 ± 39	64 ± 17	0.04–0.05	ns
2-Methylbutyl acetate	313 ^5a^; 1083 ^5b^ (fruity)	39.5 ± 5.3	50 ± 3	41.1 ± 5.6	58 ± 18	47.0 ± 10.8	0.13–0.18; 0.04–0.05	ns
3-Methylbutyl acetate	300 ^1^ (banana)	170 ± 27	277 ± 67	176 ± 42	288 ± 127	232 ± 92	0.57–0.96	ns
Total (mg L^−1^)	-	98 ± 13	102 ± 27	102 ± 14	88 ± 6	91 ± 18	-	ns
Acids								
2-Methylbutanoic acid	2200 ^2^ (cheesy)	286 ± 11	317 ± 120	317 ± 58	443 ± 104	311 ± 45	0.13–0.20	ns
3-Methylbutanoic acid	33.4 ^3^ (blue cheese)	386 ± 17	448 ± 150	433 ± 115	643 ± 180	436 ± 81	11.5–19.2	ns
2-Methylpropanoic acid	2300 ^3^ (cheese, rancid)	1324 ± 184	1292 ± 177	1439 ± 212	2000 ± 573	1470 ± 214	0.56–0.87	ns
Acetic acid (mg L^−1^)	2 ^1^ (mg L^−1^) (vinegar)	688 ± 152	669 ± 102	617 ± 156	659 ± 146	659 ± 36	0.31–0.34	ns
Octanoic acid	500 ^3^ (cheese)	1442 ± 517	1878 ± 251	1085 ± 483	1335 ± 58	1800 ± 405	2.2–3.8	ns
Decanoic acid	1000 ^3^ (rancid, fat)	417 ± 147 b	661 ± 130 a	355 ± 145 b	336 ± 22 b	536 ± 19 ab	0.34–0.66	<0.05
Total (mg L^−1^)	-	692 ± 153	673 ± 103	621 ± 157	664 ± 147	663 ± 37	-	ns
**C_13_-Norisoprenoids**								
α-Ionone	NA	1.30 ± 0.03	1.26 ± 0.05	1.35 ± 0.03	1.30 ± 0.04	1.21 ± 0.04	-	ns
β-Ionone	0.09 ^3^ (violet, floral)	1.23 ± 0.07	1.12 ± 0.06	1.27 ± 0.09	1.19 ± 0.06	0.04 ± 0.16	0.45–14	ns
β-Damascenone	0.05 ^1^ (rose, honey)	1.40 ± 0.35	0.96 ± 0.32	0.80 ± 0.21	1.41 ± 0.45	1.17 ± 0.46	16–28	ns
Total (µg L^−1^)	-	3.93 ± 0.45	3.34 ± 0.43	3.42 ± 0.33	3.90 ± 0.55	2.42 ± 1.08		ns

Different letters along the line discriminate the treatments significantly different from one another (*p* < 0.05, Duncan’s multiple range-test). ^1^ Determined in 10% *v/v* aqueous ethanol [27]; ^2^ reported in Bakker and Clarke [18]; ^3^ determined in 11% *v*/*v* aqueous ethanol with 7 g L^−1^ glycerol, at pH 3.2 [25]; ^4^ reported in Etievant [28]; ^5a^ determined in 12% *v*/*v* aqueous ethanol [29]; ^5b^ determined in model wine [29].

**Table 4:** Mean (±SD) of chromatic and colour structure measurements of 2018 Pinot noir wines (*n* = 3 per region).

Attribute	Adelaide Hills	Yarra Valley	Mornington Peninsula	Northern Tasmania	Southern Tasmania	*p*
Colour density (AU)	4.57 ± 0.68	4.02 ± 0.68	3.70 ± 1.06	4.66 ± 1.26	4.20 ± 0.20	ns
Hue	0.86 ± 0.07 a	0.75 ± 0.02 bc	0.81 ± 0.02 ab	0.72 ± 0.06 c	0.72 ± 0.03 c	<0.01
Chemical age 1	0.45 ± 0.02 a	0.34 ± 0.04 c	0.41 ± 0.02 ab	0.36 ± 0.02 bc	0.33 ± 0.04 c	<0.01
Chemical age 2	0.16 ± 0.02 a	0.11 ± 0.03 b	0.16 ± 0.02 a	0.14 ± 0.01 ab	0.11 ± 0.03 b	<0.05
Total anthocyanin (mg L^−1^)	98 ± 13 ab	114 ± 16 a	76 ± 16 b	109 ± 23 ab	131 ± 23 a	<0.05
Non-bleachable pigment (AU)	1.12 ± 0.22	0.80 ± 0.21	0.84 ± 0.29	0.99 ± 0.32	0.81 ± 0.12	ns
Total pigment (AU)	6.78 ± 0.95	7.1± 0.9	5.23 ± 1.26	7.11 ± 1.70	7.89 ± 0.96	ns
(%) Non-bleachable pigment	16.4 ± 1.6 a	11.3 ± 2.8 b	15.9 ± 2.4 a	13.8 ± 1.3 ab	10.6 ± 2.9 b	<0.05
Total phenolics (AU)	34.1 ± 5.3	33.1 ± 3.2	39.3 ± 11.2	32.9 ± 10.4	28.0 ± 4.9	ns
Total tannins (g L^−1^)	0.78 ± 0.41	0.72 ± 0.17	1.22 ± 0.63	0.87 ± 0.64	0.44 ± 0.30	ns

Different letters along the line discriminate the treatments significantly different from one another (*p* < 0.05, Duncan’s multiple range-test).

## Supplementary Materials

The following are available online at https://www.mdpi.com/2304-8158/9/9/1142/s1, Table S1: Retention times, target ions and qualifier ions of internal standards and analytes; Table S2: All sensory attributes frequency table.
