# Supplementary material for: Preliminary Study of Australian Pinot Noir Wines by Colour and Volatile Analyses, and the Pivot© Profile Method Using Wine Professionals
Source: Foods. 2020 Aug 19;9(9):1142. doi: 10.3390/foods9091142 (PMC7555393; doi:10.3390/foods9091142)
Supplement: Supplementary file 1 [file foods-09-01142-s001.pdf]

foods

MDPI

Supplementary Table S1. Retention times, target ions and qualifier ions of internal standards and analytes.

Internal Standard	RT (min)	Target Ion	Qualifier Ions	Analyte	RT (min)	Target Ion	Qualifier Ions
d_8-Ethyl acetate	4.8	96	46, 76	Ethyl acetate	5.1	88	61, 70
d_9-2-Methylpropanol	5.9	83	46, 50	2-Methylpropanol	6.1	74	41
d_3-Acetic acid	6.4	63	48, 77	Acetic acid	6.7	60	43, 45
d_{10}-Butanol	7.3	64	46, 48	Butanol	7.6	56	41, 55
d_5-Ethyl propanoate	8.6	107	76, 77	Ethyl propanoate	8.8	102	57, 75
d_5-Ethyl 2-methylpropanoate	11.0	121	93, 106	Ethyl 2-methylpropanoate	11.2	116	88, 101
d_9-3-Methylbutanol	11.0	78	60, 77	2-Methylbutanol	11.5	70	41, 57
				3-Methylbutanol	11.4	70	41, 57
d_5-Propanoic acid	11.4	79	62, 77	Propanoic acid	11.7	74	57, 73
d_9-2-Methylpropyl acetate	12	64	75, 78	2-Methylpropyl acetate	12.3	56	73, 86
d_5-Ethyl butanoate	13.9	93	106, 121	Ethyl butanoate	14.1	88	71, 101
d_7-2-Methylpropanoic acid	15.8	95	50, 77	2-Methylpropanoic acid	16.4	88	43, 73
d_5-Ethyl 3-methylbutanoate	18.29	93	120, 135	Ethyl 2-methylbutanoate	18.22	115	88
				Ethyl 3-methylbutanoate	18.5	88	85, 115
d_7-Butanoic acid	18.9	63	50, 77	Butanoic acid	18.8	60	45, 73
d_9-3-Methylbutyl acetate	20.2	79	78, 89	2-Methylbutyl acetate	21.1	55	57, 72
				3-Methylbutyl acetate	20.8	55	87, 88
d_{13}-Hexanol	21.4	78	62, 64	Hexanol	22.3	69	55, 56
d_7-3-Methylbutanoic acid	23.8	91	61, 62	2-Methylbutanoic acid	25	87	57, 74
				3-Methylbutanoic acid	24.4	87	60, 61
d_5-Ethyl hexanoate	28	93	106, 120	Ethyl hexanoate	28.1	88	99, 115
d_{13}-Hexyl acetate	28.3	96	50, 78	Hexyl acetate	28.5	84	69, 73
d_{11}-Hexanoic acid	28.9	63	77, 93	Hexanoic acid	29.7	60	73, 87
d_3-2-Phenylethanol	32	125	93, 94	2-Phenylethanol	32.1	122	91, 92
d_5-Ethyl octanoate	32.7	93	106, 177	Ethyl octanoate	32.8	88	101, 127

d_{15}-Octanoic acid	33.3	109	77	Octanoic acid	33.7	101	85, 115
d_3-2-Phenylethyl acetate	35.4	106	93, 107	2-Phenylethyl acetate	35.4	104	65, 91
d_5-Ethyl decanoate	37.7	106	162, 205	Ethyl decanoate	37.7	101	157, 200
d_{19}-Decanoic acid	37.9	63	77, 141	Decanoic acid	38.1	73	60, 172

Supplementary Table S2. All sensory attributes frequency table.

	Colour				Aroma					Palate				
	red	purple	brown	dense	floral	oaky	spice	dark fruits	red fruits	astringent	acidic	soft	red fruits	complex
AH1	7	5	10	5	6	11	8	9	8	4	4	6	7	6
AH2	2	3	10	8	5	7	7	6	5	5	5	5	5	5
AH3	4	6	7	8	8	6	10	9	6	6	4	9	4	9
YV1	5	10	6	10	7	8	11	7	6	8	4	8	6	7
YV2	8	6	7	7	5	7	7	7	6	6	5	8	7	9
YV3	9	4	7	6	7	7	6	6	4	4	5	7	7	6
MP1	5	3	7	2	5	6	7	4	9	10	9	6	8	5
MP2	6	7	7	11	8	6	8	6	6	15	8	5	4	5
MP3	7	4	6	4	7	6	10	4	10	10	7	6	7	8
NT1	7	8	6	11	9	9	7	12	4	5	4	10	6	7
NT2	6	5	8	3	5	8	11	7	6	7	8	7	6	7
NT3	6	16	3	9	4	10	7	10	7	13	9	4	5	5
ST1	5	9	6	9	5	9	10	10	4	8	3	7	7	7
ST2	4	5	5	3	9	10	3	8	5	5	5	6	7	8
ST3	7	3	7	5	8	7	8	4	9	1	7	6	8	10

AH, Adelaide Hills; YV, Yarra Valley; MP, Mornington Peninsula; NT, Northern Tasmania; ST, Southern Tasmania.
